# How Does the Accuracy of Children’s Number Representations Influence the Accuracy of Their Numerical Predictions?

**DOI:** 10.3389/fpsyg.2022.874230

**Published:** 2022-06-15

**Authors:** Bradley J. Morris, Rachael Todaro, Tracy Arner, Jennifer M. Roche

**Affiliations:** ^1^Department of Educational Psychology, Kent State University, Kent, OH, United States; ^2^Department of Psychology, Temple University, Ambler, PA, United States; ^3^Department of Psychology, Arizona State University, Tempe, AZ, United States; ^4^Department of Speech Pathology and Audiology, Kent State University, Kent, OH, United States

**Keywords:** numerical predictions, summarization, number representations, ensemble cognition, numerical cognition

## Abstract

Predictions begin with an extrapolation of the properties of their underlying representations to forecast a future state not presently in evidence. For numerical predictions, sets of numbers are summarized and the result forms the basis of and constrains numerical predictions. One open question is how the accuracy of underlying representations influences predictions, particularly numerical predictions. It is possible that inaccuracies in individual number representations are randomly distributed and averaged over during summarization (e.g., wisdom of crowds). It is also possible that inaccuracies are not random and lead to errors in predictions. We investigated this question by measuring the accuracy of individual number representations of 279 children ages 8–12 years, using a 0–1,000 number line, and numerical predictions, measured using a home run derby task. Consistent with prior research, our results from mixed random effects models evaluating percent absolute error (PAE; prediction error) demonstrated that third graders’ representations of individual numbers were less accurate, characterized by overestimation errors, and were associated with overpredictions (i.e., predictions above the set mean). Older children had more accurate individual number representations and a slight tendency to underpredict (i.e., predictions below the set mean). The results suggest that large, systematic inaccuracies appear to skew predictions while small, random errors appear to be averaged over during summarization. These findings add to our understanding of summarization and its role in numerical predictions.

## Introduction

Predictions make the environment more understandable by easing the burden on our limited cognitive capacities (Bubic et al., 2010). Predictions are generated, in part, by summarizing relevant information and using this summary as the basis for plausible forecasts (Kveraga et al., 2007; Henderson, 2017 Masnick and Morris, 2022). People rapidly summarize perceptual (e.g., color and position) and cognitive features (e.g., interpreting emotions from faces) in complex scenes (Whitney and Yamanashi Leib, 2018), which become available for predictions (Kveraga et al., 2007). Predictions are derived, in part, from summaries of individual values (e.g., approximate means from a set of numbers; Alvarez, 2011). Substantial evidence indicates that adults generate predictions close to the set mean, a reasonable indicator of future states (Irwin and Smith, 1957; Griffiths and Tenenbaum, 2006). However, children’s predictions are less accurate than adults’ though the cause of this difference is unclear (Masnick and Morris, 2008). Because predictions emerge from a summary of the statistical properties of a number set, or an implicit aggregation of individual numerical representations (Alvarez, 2011; Morris and Masnick, 2015), the accuracy of individual number representations (i.e., mapping between relative magnitude and number) should influence summary accuracy.

One open question is the extent to which estimation and prediction errors are due to imprecision in the summary values (Erlick, 1964; Peterson and Beach, 1967; Zou and Bhatia, 2021). For example, adults show a tendency to underpredict or produce predictions below the actual value (Roy et al., 2005). When asked how long it would take to complete an assignment, participants often underestimated the amount of time for completion (Koole and Spijker, 2000) and when asked to estimate how long it would take to make an origami figure, those with more experience were also more likely to underestimate their predictions (Roy and Christenfeld, 2007). Are underestimates due to inaccuracies in the individual estimates being summarized? Although there have been investigations into the cognitive mechanisms underlying the generation of predictions (Bubic et al., 2010; Henderson, 2017), there has been relatively little focus on how the accuracy of predictions is related to the accuracy of the individual representations from which they are derived.

Undoubtably, predictions should be less accurate if they are derived from highly inaccurate representations. However, little is known about the exact relation between the accuracy of individual representations and the predictions derived from them. We explored three possibilities. One possibility is that inaccurate individual representations minimally affect predictions because summaries “average over” noise to yield an estimate or prediction that is more accurate than any individual representation. This pattern is demonstrated in the wisdom of crowds in which summarizing individual representations reduces overall error (Surowiecki, 2005). In original study of Galton (1907), the individual estimates of the weight of an ox were highly variable but the distribution of estimates reduced individual error estimates, yielding a summary very close to the actual weight (within 1%). Such a pattern requires a stochastic distribution of individual values in which inaccuracies deviate randomly from the mean and their aggregation smooths over individual deviations. A second possibility is that individual values deviate non-randomly (e.g., consistently above the mean), which yields inaccurate summaries and predictions (Simmons et al., 2011). For example, the summaries from knowledgeable sports fans were inaccurate because individual fans overestimated player performance, leading to inaccurate predictions (Simmons et al., 2011). A third possibility is that both the direction and magnitude of inaccuracy will influence prediction accuracy. For example, aggregating large overestimates would likely result in an overprediction (i.e., prediction that is above the mean) while small inaccuracies might be averaged over regardless of direction.

We investigated these relations in the domain of children’s numerical predictions because young children often have less accurate number representations than adults (Kim and Opfer, 2018), which provides an opportunity to measure the degree to which their accuracy influences numerical predictions. The accuracy of number representations improves over development as symbolic and approximate number representations become aligned (Lourenco, 2016). For example, children younger than nine may mark 200 at the location for 600 on a 0–1,000 number line, indicating an inaccurate number representation (Thompson and Opfer, 2010). Number representations in the 0–1,000 range become more accurate throughout development (Schneider et al., 2017) as children analogically map magnitudes to their relative positions on a mental number line (Fitzsimmons et al., 2021).

Returning to the possibilities describe above, one possibility is that there is no relation between number line accuracy and prediction accuracy. Averaging over multiple values may control for relatively small errors in individual magnitudes (or evenly distributed errors), much like estimates from crowds average over errors in individual estimates (Herzog and Hertwig, 2014). If prediction accuracy is related to the accuracy of individual number representations, we should see non-random error patterns in number line estimation and prediction errors in that direction. For example, in the 0–1,000 range, there is evidence that children tend to overestimate individual magnitudes for smaller magnitude numbers (e.g., 150) and underestimate magnitudes for larger magnitude numbers (e.g., 800; Zax et al., 2019). If a set of numbers with an arithmetic mean of 200 are erroneously placed around the 600 position on a number line, the summary of these numbers should center (inaccurately) near 600. In such a case, overestimating the position of individual numbers should yield an overprediction (i.e., above the set mean). Finally, if there is a relation between the direction and magnitude of number accuracy and prediction accuracy, we should see prediction errors only with large inaccuracies and small (or no) errors with small inaccuracies. In this case, there should be a threshold after which relatively large errors in individual magnitudes will yield larger prediction errors. If this is the case, individual number accuracy should show no strong relation to prediction accuracy until individual number inaccuracies are relatively large, though there is currently no evidence for a precise threshold.

We investigated the relation between the accuracy of children’s number representations and the accuracy of their numerical predictions in children ages 9–13 because children in this age range should be fluent with numbers in the 0–1,000 range and because there is often lower accuracy in number representations for 9-year-old compared to older children (Booth and Siegler, 2006). To investigate these questions, we measured individual number accuracy using a number line estimation task and prediction using the Homerun Derby paradigm (Morris and Masnick, 2015).

## Materials and Methods

### Participants

An *a priori* power analysis indicated a total of 258 participants were needed to establish statistical sensitivity (repeated measures with a three factor between-subjects effect and a moderate effect size; effect size *f* = 0.14, *α* = 0.05, *1-ß* = 0.80). A total of 279 children were recruited from five public schools in Northeast Ohio. The Ohio Department of Education School Report Cards for the 2017–2018 school year indicated that 63.6% of children in these schools were economically disadvantaged. Forty-one third-graders (*M*_age_ = 9.47, *SD* = 0.50, 63% female, 34% African American, and 66% Caucasian), 71 fourth-graders (*M*_age_ = 10.35, *SD* = 0.45, 41% female, 31% African American, 55% Caucasian, 10% Latinx, and 4% Asian/Pacific Islander), 54 fifth-graders (*M*_age_ = 11.32, *SD* = 0.33, 44% female, 15% African American, 77% Caucasian, 2% Latinx, 2% Asian/Pacific Islander, and 2% Other), and 113 sixth-graders (*M*_age_ = 12.38, *SD* = 0.44, 44% female, 20% African American, 71% Caucasian, 10% Latinx, 2%, and >1% Other) participated in the experiment. Children aged 9–13 were recruited for this work because the youngest children in this age range often demonstrate lower accuracy in number representations than older children (Booth and Siegler, 2006). Because this is the first experiment to investigate these relations, this seemed a reasonable range of ages for the sample and children in this age range are typically sufficiently familiar with 0–1,000 values to complete the task. Children’s participation was completely voluntary, and experimenters informed children that they could stop participating at any time for any reason. Small rewards were provided after tasks were completed (e.g., pencils). The experimenters were Caucasian female graduate research assistants.

### Procedure and Materials

Children completed a number line estimation task (Thompson and Opfer, 2010) and a numerical prediction task (Morris and Masnick, 2015) using an offline version of the Qualtrics application on iPad minis. The order of the number line estimation and the numerical prediction tasks were counterbalanced by class such that half of the students in each grade-level received the number line estimation task first and the other half received the numerical prediction task first. Preliminary analyses indicated no order effects. The experimenters provided brief instruction on how each task would work on the iPad and what the students should do when they finished. Finally, as noted below, attention checks were included in both tasks and used as exclusion criteria prior to data analysis.

#### Number Line Estimation Task

The number line was used to measure number magnitude estimation based on the extensive literature demonstrating its reliability and relation to mathematical processing (Schneider et al., 2017). Children completed a number line estimation task that required them to estimate the placement of 22 numbers using a 0–1,000 bounded number line (Thompson and Opfer, 2010). Each number was presented directly above the 0–1,000 number line on an iPad mini. Children dragged a digital slider to where they estimated the number went on the number line. Upon introducing the task to the children, the experimenter read the directions aloud, “This is a number game. There will be a number above each number line. Your job is to move the [dot] to show where that number goes on a number line” and demonstrated how to tap the iPad screen and drag the digital slider across the number line. Children spent approximately 5–10 min completing the number line estimation task. Two attention checks were implemented (e.g., “Move the slider all the way to the left.”) to determine whether or not the children completed the task reliably. All children passed the attention checks, and their data were subject to analysis. We calculated children’s estimation accuracy by subtracting the observed number line estimate from the target number divided by 1,000 (Percent Absolute Error; Thompson and Opfer, 2010). For example, if the target number was 250 and the participant estimated 300 on the number line, the Percent Absolute Error = [250–300]/1,000 = ABS(−0.05) = 0.05 (see Table 1 for means and SEs).

**Table 1:** Means and SEs for Prediction Error and Percent Absolute Error by grade level.

Grade	PAE	PE
3	0.14(0.07)	0.13(0.47)
4	0.09(0.05)	0.05(0.41)
5	0.07(0.04)	−0.05(0.33)
6	0.08(0.05)	−0.02(0.35)

#### Numerical Prediction Task

Children completed the numerical prediction task, which required them to predict how far a baseball would travel based on players’ previous batting distances (i.e., home run derby task; based on Morris and Masnick, 2015). Before starting the task, experimenters read the following instructions aloud:

*We are going to play the Home Run Derby game. A home run derby is a contest in baseball to see which player can hit the ball the farthest and get the most home runs. You will be seeing the results from several batters in a home run derby. Each slide will show how far a player hit a series of balls in number of feet.*

On the following screen, further instructions were explained to the children:

*Your job is to tell us how far you think the player will hit the ball on the next at bat. Use the keyboard to enter a number in the space below each question. Now let us begin. Remember to tell us how far the player will hit the ball the next time.*

Experimenters demonstrated to the children how to progress through the task by completing two practice trials with them that included three distances presented simultaneously on the screen (e.g., 305, 412, and 358) for 4 s. Afterward, participants were prompted to enter their prediction on the following screen in a text response field below the prompt, “How far do you think the next ball will go?” After practice, participants completed a total of 30 trials of the numerical prediction task. Participants were not given assistance or feedback from the experimenters. Children made predictions based on five sets each of 4, 5, 8, 9, 12, and 13 distances and distances were presented simultaneously. Sets were presented from smallest to largest (i.e., 4–13) for all participants. The sets of four distances were presented for 4 s and presentation duration increased 500 ms for each additional distance in the set such that sets of 5 = 4.5 s, sets of 8 = 6 s, sets of 9 = 6.5 s, sets of 12 = 8 s, and sets of 13 = 8.5 s. Children were given an unlimited amount of time to make their prediction before moving on to the next number set and were unable to move on to the next set until they entered a response. Children completed the task within approximately 15–20 min. Two attention checks were implemented in the numerical prediction task (e.g., Type the word “star” in the box below.) to ensure participants were completing the task reliably. All participants passed the attention checks and their data were subject to analysis. Prediction accuracy was calculated by subtracting the observed prediction from the set mean divided by the set mean (i.e., Prediction Error: PE). For example, if the set mean was 200 and the observed prediction was 180, the PE = [200–180]/200 = 0.10 (see Table 1 for means by grade).

### Analytic Approach

During data cleaning and preparation, we noticed that some of the participants produced responses that were not plausible on the numerical prediction task (e.g., entering responses like 1,234,567,890 or 77,777,777). Because of this, we decided to systematically remove any data that was at least two SDs above the largest set size mean (806). This resulted in a loss of approximately 15% of the numerical prediction data (reducing the data set to 7,509 data points from 8,880 data points). There was no clear indication that the participants failed to complete the number line estimation task as accurately as possible, therefore, we did not reduce this dataset through the removal of outliers. However, three trials in the number line task were removed from the dataset because they were numerically smaller than three digits. This was done to better align the two data sets to contain only three-digit numbers.

We then analyzed each dataset (number line estimation; numerical prediction) to estimate model fit based on linear, logarithmic, and exponential transformations of the data (Opfer and Siegler, 2007). We also evaluated the two data sets using a change point analysis to determine the point at which the dependent variables (number line PAE; prediction error) changed as a function of grade level. The final analysis included a mixed random effects model that evaluated number line PAE and grade level as predictors of errors in numerical prediction. Fully maximal random effect structures permitting model convergence were always implemented, and participant and trial were set as random effects, using R (R Development Core Team, 2012). Analyses are presented below for the (1) number line dataset, (2) numerical prediction dataset, and (3) the prediction error as predicted by number line PAE by grade level. All analyses and R scripts are provided in the Open Science Framework.^1^

## Results

### Number Line Percent Absolute Error

**Figure 1:**
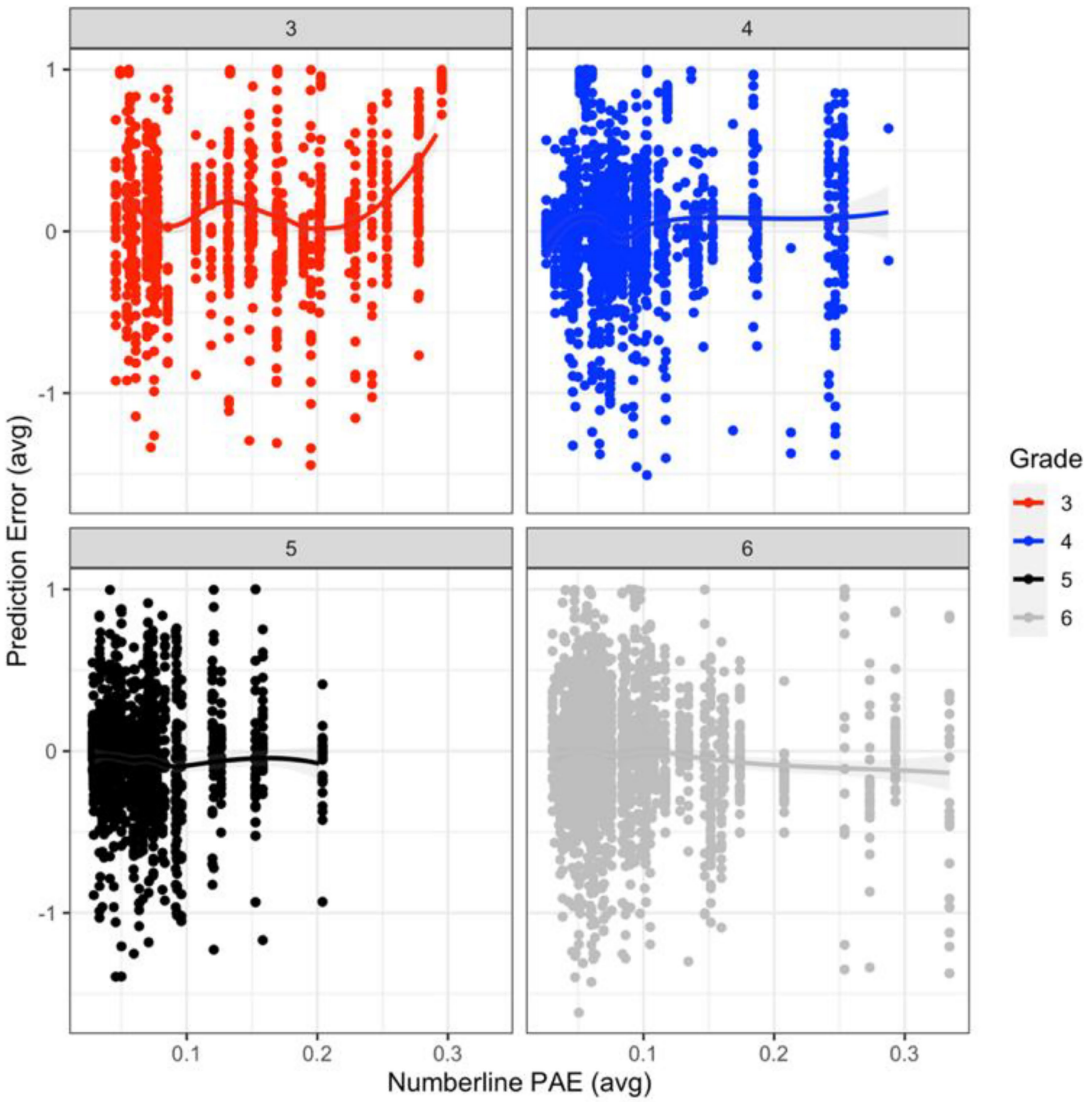
Relation between mean prediction error (PE) and number line percent absolute error (PAE), with SEs (gray shading) by grade level.

Three mixed random effects models were built to evaluate model fit based on linear (no transformation), logarithmic, and exponential transformations of the number line PAE measure by grade level (fixed effect). Results indicated that the exponential model provided the best fit to the number line PAE data [Akaike Information Criterion (AIC) = −233.30, *x*^2^ = 544.87, *p* < 0.001]. Evaluation of the exponential model indicated a significant relation between age and number line PAE (*ß* = −0.31, *SE* = 0.03, *t* = −11.98, *p* < 0.001, and *R*^2^ = 0.55). This suggests that inaccuracy for third graders was not random, number line PAE decreases as age increases, and age accounted for approximately 55% of the variance in number line PAE (Figure 1).

### Prediction Error

Three mixed random effects models were built to evaluate model fit based on linear (no transformation), logarithmic, and exponential transformations of the Prediction Error (PE) measure by grade level (fixed effect). However, the logarithmic model was dropped from the analysis because the model failed to converge and NaNs (i.e., not a number) were produced. Evaluation of the linear (non-transformed) and exponential models indicated that the linear model produced the best fit, because it had the smallest AIC (AIC = −3303.4), but the linear and exponential models did not have significantly different model fits. Since there was no significant difference between the models, and the linear model produced the smallest AIC, it was retained and is interpreted. The results of the non-transformed PE measure indicated a significant relation between age and PE, such that decreases in PEs occurred as grade level increased (*ß* = −0.11, *SE* = 0.03, *t* = −3.91, *p* < 0.001, and *R^2^* = 0.64). Age accounted for approximately 64% of the variance in PE (Figure 1).

### Prediction Error Predicted by Number Line PAE

**Figure 2:**
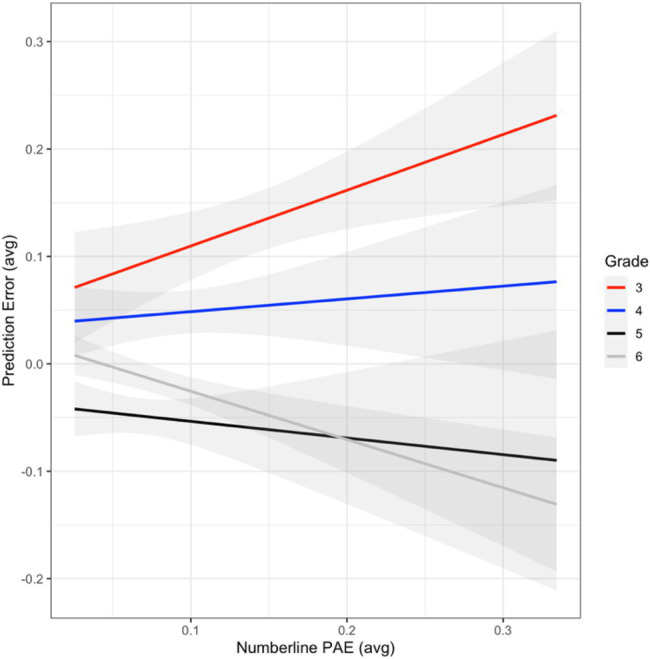
Smoothed (using stat_smooth = glm) PE (prediction error) measure (non-transformed) predicted by number line PAE and grade.

A mixed random effects model was used to evaluate the non-transformed PE measure as a function of number line PAE (exponential transformation) by age. The results from this model indicated a significant main effect of age (*ß* = 0.55, *SE* = 0.23, *t* = 2.37, and *p* = 0.02), and a significant interaction between age and number line PAE (*ß* = −0.60, *SE* = 0.21, *t* = −2.86, and *p* = 0.004), with age, number line PAE, and their interaction accounting for approximately 33% of the variance in PE. The interaction between age by number line PAE indicated differences only existed between the youngest (i.e., third graders) and oldest children (i.e., sixth graders) in our sample. A test of the simple effects indicated that third graders produced larger prediction errors relative to sixth graders (*ß* = −0.78, *SE* = 0.31, *t* = −2.53, and *p* = 0.01; mean difference = 0.12). Third graders had a significantly stronger positive relation between number line PAE and PE than sixth graders (i.e., as number line PAE increased, PE also increased for third graders; *ß* = 0.82, *SE* = 0.27, *t* = 3.00, and *p* = 0.003; mean difference = 0.12; see Figure 2).

Because of this difference, and to align with the previous two analyses, participants’ data were evaluated based on change points within the number line PAE and PE measures. Only third graders’ data showed a significant change point between PE and number line PAE. Specifically, the results from the change point analysis indicated a significant change point at 0.31 (number line PAE) and 0.01 (PE) for third graders (*θ* = 1.31, *α* = 0.01, *p* = 0.048; 95% CI = [1.06, 1.12]; see Figure 4, supplementary materials in the OSF site for more details regarding the break point data; see footnote 1).

## Discussion

Predictions are generated, in part, by summarizing over multiple, individual items (Kveraga et al., 2007; Henderson, 2017). These summaries constrain candidate predictions because the range of past values sets a reasonable guide for future forecasts (representativeness; Birnbaum, 1976; Griffiths and Tenenbaum, 2006). One gap in the literature is how the accuracy of individual representations affects summaries and in turn, influences subsequent predictions. Previous research has investigated the influence of underlying representations on predictions (e.g., the role of memory for previous events when estimating time; Roy et al., 2005); however, there has been little systematic investigation in the domain of numerical predictions. We investigated this question in the domain of children’s numerical predictions because young children’s numerical representations are initially inaccurate, typically demonstrating underestimates for larger, individual values (Fitzsimmons et al., 2021).

We measured the accuracy of children’s number representations using a number line and the accuracy of their numerical predictions by comparing their predictions to the mean of a number set. Because number sets are summarized, children with less accurate number representations generated less accurate predictions suggesting that accuracy was not due to deficits in younger children’s mathematics knowledge. Overall, individual number representation accuracy and age accounted for 33% of the variance in children’s numerical prediction accuracy, similar to the proportion of variance explained by number representation in children’s overall mathematical performance (0.30 effect size; Schneider et al., 2017). Because nearly all of the variance was from third graders’ low accuracy, these data and the change point analysis suggest that the accuracy of third graders’ individual number representations influenced the accuracy of their summaries of set properties (e.g., mean; Alvarez, 2011). These data suggest that the direction and magnitude of inaccuracies in individual number representations influenced numerical predictions. This was most evident for third graders, who demonstrated the least accurate individual number representations (i.e., higher PAE), which were associated with the least accurate predictions (i.e., higher PE). Interestingly, third grader’s inaccuracies were systematic *overestimates* in number magnitude, and the change point analysis indicated that the larger the deviation in individual number representations (PAE > 0.31), the larger the overprediction error. Older children produced more accurate individual number representations and smaller, randomly distributed errors in numerical prediction. Underestimating predictions was not related to underestimate individual number representations. In fact, underestimations of predictions were more frequent for children with more accurate individual number representations, similar to the results of Roy and Christenfeld (2007).

This experiment is not without limitations. One limitation is that the findings are correlational, rather than causal. Although correlational, these findings suggest that improvements in number representation accuracy drive initial improvements in predictions, likely due to progressive alignment of relative magnitudes (Thompson and Opfer, 2010). Another potential limitation is that the use of the number line task has been criticized for being unrelated to mental representations of relative magnitude (Slusser and Barth, 2017). Future research should include other measures of magnitude representation (e.g., magnitude comparisons) to address this possibility. Future research should investigate the influence of other number features (e.g., perceptual; Cohen, 2009) and the interaction between prior knowledge (Griffiths and Tenenbaum, 2006) and number representations. The results suggest that developmental improvements in number representation may be a significant factor driving initial improvements in prediction accuracy. That said the results from older children demonstrate that while underlying number representations are important, they are only part of a larger story.

Our results illustrate the complex influence of underlying representations on predictions. Predictions are an attempt to generate a forecast beyond the information given but are constrained by available information. Predictions depend on summaries of current representations; thus, the accuracy of these representations influences the accuracy of the predictions in specific ways. Our results suggest that third grader’s overestimates of individual numbers, often large in magnitude, were associated with overpredictions. Consistent with previous research with adults, older children, who demonstrated accurate individual number representations, showed a pattern of underpredicting or making predictions lower than the set mean (Roy et al., 2005). This suggests a complex developmental pattern in which inaccuracy in individual representations is initially highly disruptive to predictions. It also suggests multiple avenues for future research that investigate developmental changes (e.g., changes in working memory capacity) as well as individual differences (e.g., changes in strategy use). For example, increased accuracy of individual representations and higher-level processes (e.g., strategy use) might influence summary values generated to make sense of number sets. Another future direction is to further investigate the similarities and potential differences in the processes underlying estimation and prediction. This is relevant to our findings because prediction might require greater cognitive resources (e.g., working memory) than estimation, which may help to explain developmental differences in performance. Finally, future research on predictions should include measurement of underlying representations to better understand their role in shaping the accuracy of predictions, particularly how they evolve across development.

## Data Availability Statement

The analyses and R Scripts used in this study are available in the public domain and can be accessed via The Open Science Framework (https://osf.io/856zm/).

## Ethics Statement

The studies involving human participants were reviewed and approved by Kent State University IRB. Written informed consent to participate in this study was provided by the participants’ legal guardian/next of kin.

## Author Contributions

BM was the lead in the planning, study design, and took primary responsibility for drafting and writing the manuscript for publication. RT and TA were the leads for data collection. JR was the lead for data analysis and reporting and interpretation of the results. All authors contributed to the article and approved the submitted version.

## Conflict of Interest

The authors declare that the research was conducted in the absence of any commercial or financial relationships that could be construed as a potential conflict of interest.

## Publisher’s Note

All claims expressed in this article are solely those of the authors and do not necessarily represent those of their affiliated organizations, or those of the publisher, the editors and the reviewers. Any product that may be evaluated in this article, or claim that may be made by its manufacturer, is not guaranteed or endorsed by the publisher.

## Footnotes

^1^ https://osf.io/856zm/
